# The introduction of telemedicine is required immediately in Cambodia: Barriers and lessons from COVID-19

**DOI:** 10.7189/jogh.11.03047

**Published:** 2021-03-27

**Authors:** Buntongyi Nit, Yurie Kobashi, Sopanha Vory, Soradet Lim, Sopanharith Chea, Shunichiro Ito, Masaharu Tsubokura

**Affiliations:** 1 Department of Medicine, University of Puthisastra, Sangkat Boeung Raing, Phnom Penh, Cambodia; 2 Department of Radiation Health Management, Fukushima Medical University School of Medicine, Fukushima City, Fukushima, Japan; 3 LEBER Co., Ltd., Takano, Tsukuba city, Ibaraki, Japan

The spread of coronavirus has been rampant worldwide. In this context, telemedicine is particularly vital because it can provide medical services with minimized transmission risk for the medically or socially vulnerable and those lacking ready access to providers [1]. It has been reported that the COVID-19 outbreak has fostered the rise of telemedicine especially in developed countries [2]. However, there is little information on its progress in developing countries.

Cambodia in Southeast Asia is classified as a low and middle-income country (LMIC) by the World Bank. Moreover, the number of people infected with COVID-19 was 288 on 28 October 2020 as per the Coronavirus Resource Center 2020, Johns Hopkins University of Medicine [3]. However, the number of doctors is estimated to be 8 nurses and 2.4 doctors per 10 000 people in Cambodia [4], which is the lowest in Southeast Asia. Since medical resources are limited in Cambodia, preventing the spread of pandemics is vital; therefore, the government initiated the following at the start of 2020. First, infected patients were gathered, examined, and treated in specific hospitals that had personal protection equipment. Second, the Khmer New Year celebrations in the third week of April were postponed, and a short-term lockdown was implemented in this period to prevent the most active migration of the year. Third, mass education on the wearing of masks, alcohol sanitizing of hands, and the overall crisis was conducted through the media and social networking service (SNS). Nevertheless, the introduction of telemedicine in Cambodia is vital in preparing for the possibility of a large COVID-19 outbreak to address the issues of having an inadequate workforce and poor knowledge toward disease and health care among residents. In the present article, we discuss the current situation, the barriers it creates, and prospects regarding telemedicine development under the influence of the COVID-19 pandemic in Cambodia, as an example of Asian LMICS.

Telemedicine among residents has not spread widely in Cambodia. There is a specific gap when it comes to the availability of digital skills. Of those with tertiary education, only 32.4% use computers and the Internet, which is very low compared to 87.8% in Indonesia and 89.7% in Thailand [5]. Furthermore, according to the Cisco global digital readiness index in 2019, the digital readiness index of Cambodia was 9.27 and categorized as in the accelerating stage without enough readiness in terms of the digital system [6]. Furthermore, the ranking of Cambodia in the Global Cybersecurity Index was 92 in 2017, which is classified as the initiating stage [7]. Regarding telemedicine in Cambodia, some private clinics based overseas have only just started teleconsultation services for patients after the COVID-19 outbreak. This recent start, the high price barrier for overseas doctors, and limited availability to their existing patient networks has led to very low participation. Further the guidelines for telemedicine and for safety of medical informatics systems used in hospitals and telemedicine companies has not been published; the protection of personal information online is an important issue. To cope with this problem, the creation of regulations and guidelines that those who intend to advance telemedicine must use is crucial. Additionally, Cambodia has other barriers to technology adoption including the ease of doing business, human capital development, business and government investments, basic human needs, and start-up environments. Thus, until these components are more developed, telemedicine advancement is very difficult to realize.

**Figure:**
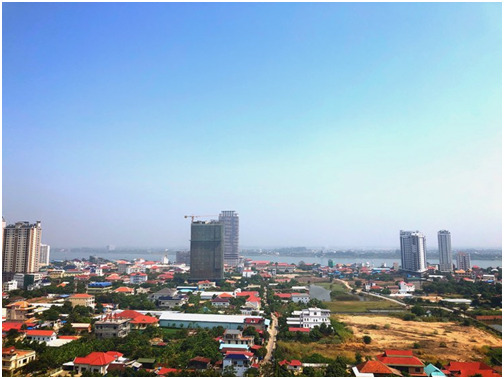
Photo: The view of Phnom Penh (from the authors’ own collection, used with permission).

Regarding the availability of digital skills, the reduction of disparities in terms of utility of network systems between urban and rural areas is essential. In Cambodia, approximately 99% of the lowest wealth quintile residents live in rural area [8]. These residents face a barrier regarding internet use as well, because the covered reception areas are restricted to urban areas. Consequently, digital infrastructure might be the one of the most productive investment approaches in rural areas. Regarding ways to ensure that government-led legislation passes including the establishment of guidelines, referring to overseas and neighboring country’s approaches might be considerable. Many developed countries’ governments have established guidelines that should be considered when conducting telemedicine. For instance, in Japan, the Ministry of Public Management, the Ministry of Economy, and the Ministry of Health have jointly issued two guidelines, which stipulate the handling of personal information and the cloud. However, the required caution regarding personal information protection or ethics issues differ for each platform type: consultation between doctors and patients, consultation between doctors, focus on only medical advice, or provision of diagnosis and treatment. Thus, under the influence of the COVID-19 pandemic, cooperation between the government and telemedicine companies has been undertaken in developed countries and some LMICs. The Cambodian government has also been making such efforts (eg, a tie-up with a foreign telemedicine company); however, guidelines have not been stipulated, and the extent to which a company can venture into telemedicine may be restricted. Regarding other barriers, fostering corroboration networks between companies and medical sectors in both the domestic and international sectors might be required. Consequently, many professionals should be hired to push the telemedicine industry forward.

The aforementioned barriers limit the widespread use of telemedicine, which might be a suitable system for improving medical care in Cambodia. Meanwhile, despite the education sector’s swift adaptation to digital platforms throughout Cambodia due to COVID-19, some problems have remained. Moreover, knowledge about COVID-19 and residents' awareness increased – factors that can help suppress a pandemic – through media and digital platforms: short messages were sent to smartphones and information was spread on TV, radio, and SNS. Subsequently, both urban and rural residents, who did not have adequate knowledge of non-communicable and other common diseases, changed their behavioral habits; this included wearing masks even under the scorching sun. Furthermore, improving the knowledge of non-doctor medical professionals in remote areas such as health centers is vital. As a solution, telemedicine is a robust method to share medical knowledge with doctors when diagnosis or judgments are difficult. Awareness that Cambodia has adequate time to introduce telemedicine might be required. Further, an optimum telemedicine platform should be selected to increase the knowledge of residents and medical professionals.

In Cambodia, unlike other countries, preparation for telemedicine introduction is required immediately. Besides, further report gathering from developing countries on telemedicine is required in the COVID-19 era.
